# Reproducibility and across-site transferability of an improved deep learning approach for aneurysm detection and segmentation in time-of-flight MR-angiograms

**DOI:** 10.1038/s41598-024-68805-w

**Published:** 2024-08-13

**Authors:** Marius Vach, Luisa Wolf, Daniel Weiss, Vivien Lorena Ivan, Björn B. Hofmann, Ludmila Himmelspach, Julian Caspers, Christian Rubbert

**Affiliations:** 1 Department of Diagnostic and Interventional Radiology, Medical Faculty and University Hospital Düsseldorf, Heinrich-Heine-University Düsseldorf, Moorenstraße 5, 40225 Düsseldorf, Germany; 2 Department of Neurosurgery, Medical Faculty and University Hospital Düsseldorf, Heinrich-Heine-University Düsseldorf, Düsseldorf, Germany; 3 Heine Center for Artificial Intelligence and Data Science (HeiCAD), Heinrich-Heine-University Düsseldorf, Düsseldorf, Germany

**Keywords:** Intracranial aneurysm, Magnetic resonance angiography, Deep learning, Convolutional neural network, Reproducibility, Brain imaging, Magnetic resonance imaging

## Abstract

This study aimed to (1) replicate a deep-learning-based model for cerebral aneurysm segmentation in TOF-MRAs, (2) improve the approach by testing various fully automatic pre-processing pipelines, and (3) rigorously validate the model’s transferability on independent, external test-datasets. A convolutional neural network was trained on 235 TOF-MRAs acquired on local scanners from a single vendor to segment intracranial aneurysms. Different pre-processing pipelines including bias field correction, resampling, cropping and intensity-normalization were compared regarding their effect on model performance. The models were tested on independent, external same-vendor and other-vendor test-datasets, each comprised of 70 TOF-MRAs, including patients with and without aneurysms. The best-performing model achieved excellent results on the external same-vendor test-dataset, surpassing the results of the previous publication with an improved sensitivity (0.97 vs. ~ 0.86), a higher Dice score coefficient (DSC, 0.60 ± 0.25 vs. 0.53 ± 0.31), and an improved false-positive rate (0.87 ± 1.35 vs. ~ 2.7 FPs/case). The model further showed excellent performance in the external other-vendor test-datasets (DSC 0.65 ± 0.26; sensitivity 0.92, 0.96 ± 2.38 FPs/case). Specificity was 0.38 and 0.53, respectively. Raising the voxel-size from 0.5 × 0.5×0.5 mm to 1 × 1×1 mm reduced the false-positive rate seven-fold. This study successfully replicated core principles of a previous approach for detecting and segmenting cerebral aneurysms in TOF-MRAs with a robust, fully automatable pre-processing pipeline. The model demonstrated robust transferability on two independent external datasets using TOF-MRAs from the same scanner vendor as the training dataset and from other vendors. These findings are very encouraging regarding the clinical application of such an approach.

## Introduction

Unruptured intracranial aneurysms occur in about 1–2% of the population^1^ and are common incidental findings in brain imaging studies, especially those including magnetic resonance angiograms (MRAs)^2^. Since aneurysm rupture might be fatal or lead to severe disability, reliable detection of unruptured aneurysms is essential^3^. However, reliably detecting small, unruptured aneurysms is challenging, especially for (sub)specialists other than neuroradiologists^4–6^. In this context, a computer-assisted approach was shown to be helpful^7^.

Deep-learning-based automatic aneurysm detection and segmentation in time-of-flight MRAs (TOF-MRAs) has been proven technically feasible^8–10^. However, models are typically trained and validated on small, homogeneous samples and there is often only limited knowledge about their generalizability, specifically the transferability to different sites and image acquisition environments—a critical factor for real-world usage^11^.

This study aimed to (1) replicate the core principles of a previous publication on automatic cerebral aneurysm segmentation in TOF-MRAs published by Sichtermann et al.^8^. Furthermore, we addressed aspects outside the scope of the previous publication by (2) providing novel, robust, more automatable pre-processing, (3) evaluating the model in patients without aneurysms and (4) critically assessing the model’s generalizability and across-site transferability using external datasets. The model was trained on locally acquired TOF-MRAs using MRI scanners from a single vendor, and the pre-processing methods comprised both the previously proposed approaches and improved, more robust and more automatable techniques. The model’s generalizability and transferability were then evaluated on both an external same-vendor and an other-vendor test-dataset obtained from independent centers, including patients with and without aneurysms.

## Methods

The requirement of informed consent was waived by the ethics committee of the Medical Faculty of the Heinrich-Heine-University Düsseldorf, Germany due to the retrospective nature of the study. All research has been performed in accordance with the relevant guidelines and regulations, including the Declaration of Helsinki. The study protocol was approved by the ethics committee of the Medical Faculty of the Heinrich-Heine-University Düsseldorf, Germany.

### Dataset

All 3D-TOF-MRAs acquired between 06/2006 and 12/2019 with ≥ 1 untreated saccular aneurysm from the local PACS were included. The examinations included patients imaged at the local tertiary care center and patients referred for consultation or treatment. All MRAs were reviewed for appropriate image quality by a neuroradiologist with ten years of experience (JC) as well as a radiologist with five years of experience (MV). Patients with a history of previous subarachnoid hemorrhage or cerebral aneurysm treatment were excluded.

**Figure 1:**
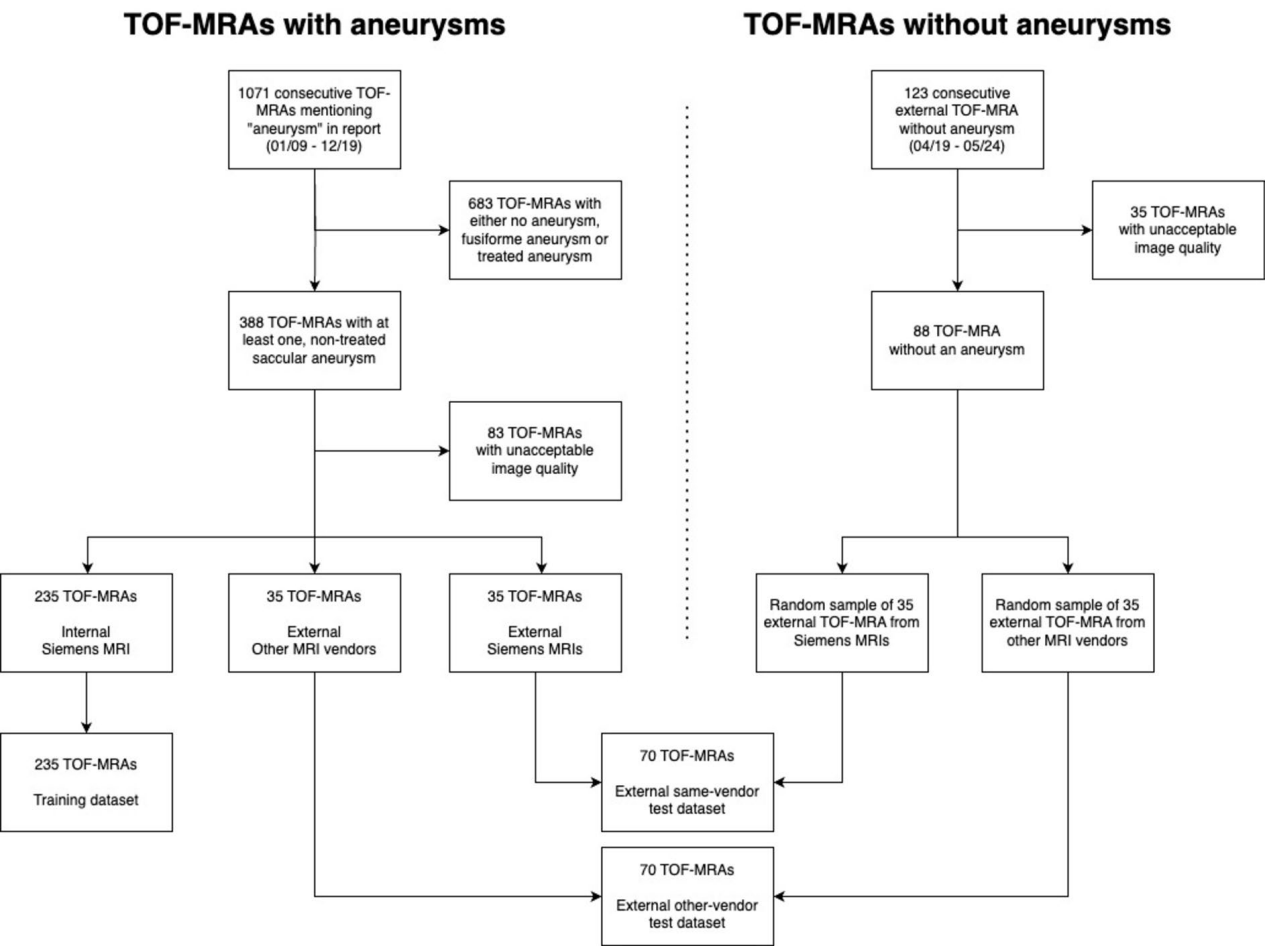
Flow diagram of the dataset selection process for the training and test data. The model was trained on 235 TOF-MRAs from MRIs of a single vendor at our institution. The model was tested on an external same-vendor dataset comprised of 70 TOF-MRAs containing images from MRI scanners of the same vendor acquired at independent institutions. The external other-vendor dataset consisted of 70 TOF-MRAs from other MRI vendors (19 GE, 46 Philips, 4 Hitachi, 1 Canon).

The final dataset included examinations acquired at the local institution and various independent centers. The examinations from the local institution were all acquired on Siemens MRI-scanners (Siemens Healthineers, Forchheim, Germany) and only included TOF-MRAs with aneurysms. These “internal” scans were used for training. Any examination acquired outside the local center was assigned to an external test-dataset, which was split into (1) external examinations acquired on a scanner by Siemens Healthineers (“external same-vendor test-dataset”) and (2) external examinations acquired on a scanner by any other vendor (“external other-vendor test-dataset”, Fig. 1). Finally, both external test-datasets were extended to include a matching number of external same-vendor or other-vendor TOF-MRAs without any aneurysms (see Fig. 1).

Voxel-wise ground-truth segmentation of the aneurysms was carried out using ITK-SNAP^12^ by a radiology resident with three years of experience in brain imaging (LW). All segmentations were reviewed by a senior neuroradiologist (JC).

### Data pre-processing

**Figure 2:**
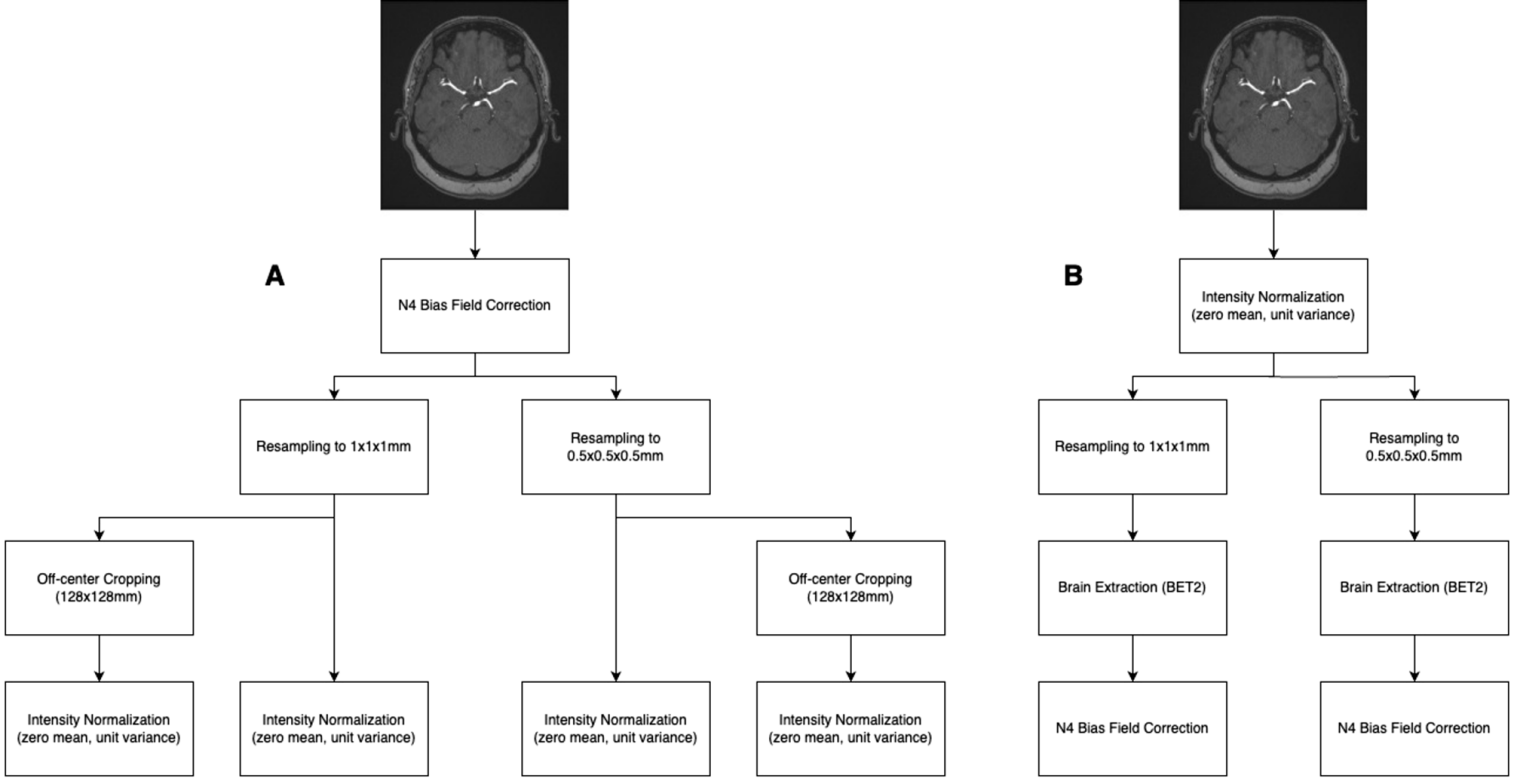
Flow-charts depicting the different preprocessing pipelines. (**A**) shows our fully-automated approaches (**B**) resembles the adapted preprocessing pipelines of a previous publication (Sichtermann et al.^8^, pipeline “B” with additional N4 bias field correction from Sichtermann et al. pipeline “D”).

Different pre-processing pipelines were applied (Fig. 2A). Each pipeline included N4 bias field correction of the whole image volume using the Advanced Normalization Tools^13^ as the first step. Then, TOF-MRAs were resampled to either 1 × 1 × 1 mm or 0.5 × 0.5 × 0.5 mm isovolumetric voxel size. In an optional additional pre-processing step, images were cropped to 128 × 128 mm in the axial plane using FMRIB’s Software Library (FSL)^14^ (left–right centered and anteriorly offset by a third of the image length to fully include the anterior circulation). The z-dimension remained unchanged. Each image volume was intensity normalized to zero mean and unit variance (according to^15^).

Furthermore, we tested a pipeline matching the pre-processing pipeline “B” from Sichtermann et al.^8^, and extended it with bias field correction as proposed in pipeline “D” (Fig. 2B), i.e. (1) intensity normalization, (2) resampling, extending the group’s approach by including 1 × 1×1 mm, (3) skull stripping using FSL’s Brain Extraction Tool (BET2, using a constant fractional intensity threshold of 0.2)^16^, and (4) concluding with N4 bias field correction^8^. We chose to extend pipeline “B” with the bias field correction of pipeline “D” to optimally reproduce the group’s core principles, while allowing for fully automated pre-processing. Pipelines “C” and “D” required volume-wise manual adjustments to BET2’s fractional intensity threshold, making automation impossible.

The previous publication explored different volume detection thresholds during post-processing to lower the false positive rate^8^. Since applying a volume threshold to the segmentation results could lead to missing small aneurysms below the detection threshold, therefore lowering sensitivity, we chose to not apply any post-processing.

### Model training and evaluation

Following the approach proposed by the previous publication^8^, a convolutional neural network (CNN) was trained separately without any pre-processing and for each pre-processing pipeline to automatically segment the aneurysms using the Open Source DeepMedic framework (https://deepmedic.org/)^15^^.^

**Figure 3:**
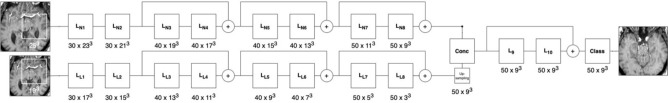
Architecture of the “DeepMedic” convolutional neural network. The network consists of two 11-layer pathways working with different resolutions of the image volume (adapted from Kamnitsas et al. and Sichtermann et al.^8,15^).

DeepMedic is a multiscale 3D CNN for voxel-wise classification of medical images, developed initially for brain lesion segmentation. It consists of a deep CNN with two pathways with 11 layers each (see Fig. 3) and has been described in more detail before^15^.

The training parameters proposed by Sichtermann et al. and Kamnitsas et al. were fully adopted^8,15^. The batch size was 10. An initial learning rate of 10^−3^ was used and gradually reduced. Nesterov Momentum was set to 0.6. L1 = 10^−6^ and L2 = 10^−4^ regularization and a dropout rate of 0.5 were used to prevent overfitting. Rectified Linear Unit activation functions and batch-normalization were used to stabilize the training. The training of each model took 6 h on an NVIDIA A100 GPU (40 GB) with 35 epochs.

### Image analysis

The trained neural network was used on both external test-datasets. Results were compared to the ground truth to calculate Dice’s similarity coefficient (DSC). A connected component (CC) analysis was performed to obtain each aneurysm separately from the segmentation results. Any overlap between a ground truth aneurysm and an automatically segmented aneurysm was considered a true positive finding. Any CC without overlap with an aneurysm in the ground truth segmentations was considered a false positive. In contrast, every manually segmented aneurysm in the ground truth without an overlapping CC was designated a false negative. Aneurysm-level sensitivity, specificity, false positive, and false negative rates were calculated on these allocations. Subgroup analysis was carried out for aneurysm location and MRI scanner field strength. The total aneurysm volume per patient was obtained from the manual segmentations to investigate any correlation with the DSC.

### Statistical analysis

The DSC between the external same-vendor and external other-vendor test-datasets was compared using an unpaired, two-sided Student’s t-test. Correlations between aneurysm size and DSC were calculated using Pearson’s correlation coefficient. The false positive rates were compared using the Mann-Whitney U test. A McNemar test was used to compare the sensitivities of two different pre-processing pipelines. To compare the sensitivities of one model between the same-vendor and the other-vendor test set, the chi-squared test was used. Continuous variables are presented as mean ± standard deviation (SD). Discrete variables are presented as “n (percent)”. Statistical analysis and image analysis to obtain the DSC and the false positive and false negative rates were performed using Python and the library “SciPy”^17^. p < 0.05 was considered statistically significant.

## Results

### Dataset

**Figure 4:**
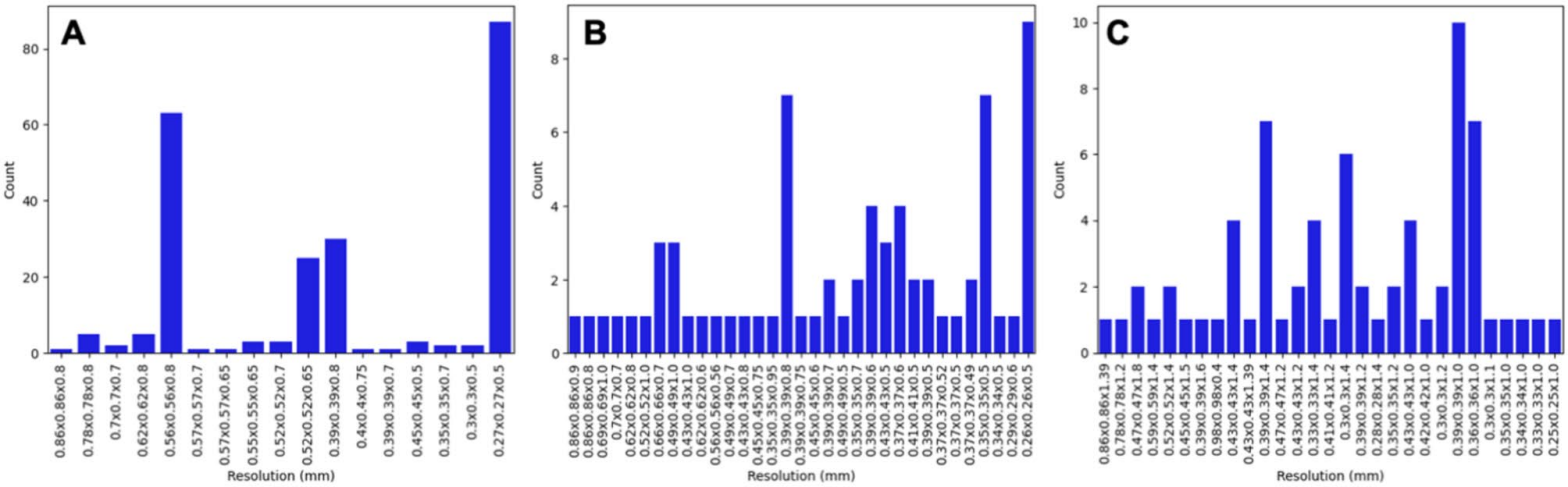
Distribution of native image resolutions in the training (**A**), same vendor (**B**) and other-vendor (**C**) dataset before resampling, sorted by voxel volume.

**Figure 5:**
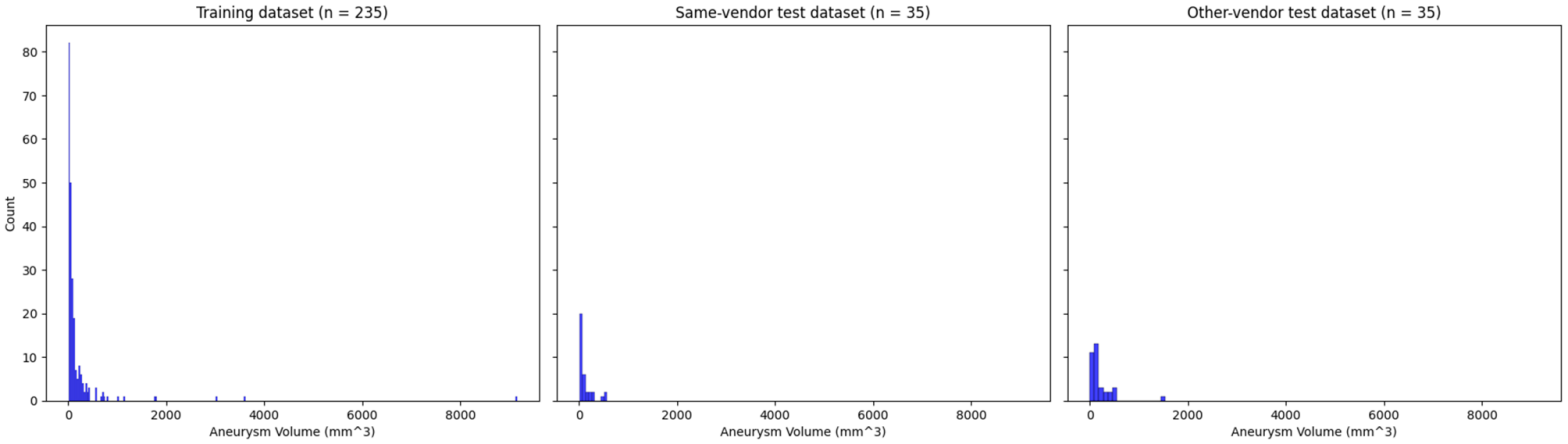
Distribution of ground-truth aneurysm volumes (in mm^3^) in the training, same-vendor and other-vendor test datasets.

In total, TOF-MRAs of 375 patients with 330 aneurysms were included (58 ± 16 years, 262 (70%) women). 305 MRAs were acquired on Siemens MRI scanners, of which 235 (77%) were acquired at the local institution and used in the training dataset (59 ± 16 years, 175 (74%) women). The other 70 MRAs performed on Siemens MRI scanners (11.5%) were acquired at other centers and were included in the external same-vendor test-dataset (59 ± 16 years, 40 (57%) women). The external other-vendor test-dataset (n = 70, 11.5%, 47 (67%) women) comprised examinations acquired on scanners from four different vendors (Table 1). The sequence parameters are summarized in Table 2 and Fig. 4 shows the distribution of image resolutions in the training, same-vendor and other-vendor dataset before resampling. Table 3 includes the distribution of aneurysms in the datasets. According to the manual segmentation, the median aneurysm volume was 69 mm^3^ (range 4–9150 mm^3^, inter-quartile-range 33–160 mm^3^, see Fig. 5).

**Table 1:** Number of patients (TOF-MRAs) in the dataset grouped by the dataset, MRI vendor, and MRI model name (magnetic field strength in brackets).

MRI vendor	Model name	# of examinations	
		With aneurysms	Without aneurysms
Training dataset (n = 235)			
Siemens healthineers	3 T (n = 120 (51%))		
	Skyra (3 T)	87	–
	TrioTim (3 T)	33	–
	1.5 T (n = 115 (49%))		
	Avanto (1.5 T)	73	–
	Sonata (1.5 T)	36	–
	Avanto fit (1.5 T)	3	–
	Magnetom sola (1.5 T)	2	–
	Magnetom vision (1.5 T)	1	–
External same-vendor test dataset (n = 70)			
Siemens healthineers	Avanto (1.5 T)	12	2
	Aera (1.5 T)	3	9
	Skyra (3 T)	2	6
	Symphony (1.5 T)	7	0
	SymphonyTim (1.5 T)	3	3
	Lumina (3 T)	0	4
	Amira (1.5 T)	1	2
	Symphony Vision (1.5 T)	2	0
	Essenza (1.5 T)	0	2
	Avanto fit (1.5 T)	1	1
	Espree (1.5 T)	1	1
	Altea (1.5)	0	2
	Verio (3 T)	1	1
	Magnetom Vision (1.5 T)	1	0
	Spectra (3 T)	1	0
	Sempra (1.5)	0	1
	HarmonyExpert (1.5 T)	0	1
External other-vendor test dataset (n = 75)			
GE healthcare (n = 19)	Signa HDxt (1.5 T)	8	2
	Signa excite (1.5 T)	2	0
	Optima MR360 (1.5 T)	2	0
	Signa artist (1.5 T)	0	2
	Genesis signa (1.5 T)	1	0
	Signa voyager (1.5 T)	1	0
	Signa explorer (1.5 T)	0	1
Hitachi medical corporation (n = 4)	Echelon (1.5 T)	3	0
	Oasis (1.2 T)	0	1
Philips medical systems (n = 46)	Achieva (3 T n = 2; 1.5 T n = 14)	9	7
	Intera (1 T n = 2; 1.5 T n = 4)	6	0
	Ingenia (1.5 T n = 8; 3 T n = 4)	3	9
	Panorama HFO (1 T)	0	1
	Achieva dStream (1.5 T n = 1; 3 T n = 1)	0	2
	Prodiva CX (1.5 T)	0	1
	Ingenia elition X (3 T)	0	8
Canon medical systems (n = 1)	Orian (1.5 T)	0	1

The external other-vendor test-dataset comprised examinations acquired on Philips (Philips Medical Systems Europe, Best, The Netherlands), GE (GE Healthcare, Chicago, Illinois), Hitachi system (Hitachi Medical Systems Europe, Metzingen, Germany) and Canon MRI system (Canon Medical Europe, Zoetermeer, The Netherlands).

**Table 2:** Median and range of the flip angle, echo time (TE) and repetition time (TR) of the time-of-flight magnetic resonance angiographies (TOF-MRAs) in the different datasets.

	Training dataset	Same-vendor test dataset	Other-vendor test dataset
Flip angle (°)	18 (14–25)	25 (14–30)	20 (18–35)
TE (ms)	3.43 (3.43–7.2)	7 (3.43–9.45)	6.5 (2.5–10.36)
TR (ms)	23 (21–37)	25 (20–47)	23 (17–44)

The datasets include TOF-MRAs with a wide range of sequence parameters acquired on MRI scanners from different vendors and with different magnetic field strengths.

**Table 3:** Aneurysms distribution for the training dataset (TOF-MRAs of Siemens Healthineers MRI scanners at the local center), as well as Dice coefficient scores (DSC) and aneurysms distribution for the same-vendor (TOF-MRAs of Siemens Healthineers MRI scanners at independent hospitals) and the other-vendor test-dataset (TOF-MRAs of different vendor MRI scanners at independent hospitals) based on the aneurysm location.

Aneurysm location	Number of aneurysms in the training dataset	DSC (number of aneurysms) external same-vendor test dataset	DSC (number of aneurysms) external other-vendor test dataset
ACA*	64 (26%)	0.67 ± 0.22 (n = 9, 24%)	0.76 ± 0.18 (n = 9, 19%)
ACI^§^	79 (32%)	0.51 ± 0.28 (n = 12, 31%)	0.56 ± 0.31 (n = 8, 17%)
MCA^$^	79 (32%)	0.64 ± 0.31 (n = 8, 21%)	0.60 ± 0.30 (n = 14, 30%)
Posterior^&^	23 (10%)	0.62 ± 0.20 (n = 9, 24%)	0.77 ± 0.12 (n = 16, 34%)
Number of aneurysms	245 (100%)	38 (100%)	47 (100%)

There was no statistically significant difference between the DSC at the different locations (p = 0.52).

*ACA anterior cerebral artery (including anterior communicating artery).

^§^*ACI* internal cerebral artery (including carotid-T and ophthalmic artery).

^$^*MCA* middle cerebral artery.

^&^posterior arteries including vertebral, basilar, cerebellar and posterior communicating arteries).

### Model performance

The model’s performances are presented in Table 4. The approach without any pre-processing yielded a high sensitivity, comparable to those after pre-processing, but a very high false-positive rate, especially in the external other-vendor test dataset. The pre-processing approach with bias field correction, a voxel size of 1 × 1 × 1 mm and cropping showed the highest DSC, highest specificity and lowest false positive rate (0.60 ± 0.25, 0.38, and 0.87 ± 1.35 FPs/case, respectively) on the external same-vendor as well as on the external other-vendor test set (0.65 ± 0.26, 0.53, and 0.96 ± 2.38 FPs/case, respectively). Sensitivity of the model was 0.97 and 0.92, respectively, with the best models achieving a sensitivity of 0.98 in the external same-vendor test-dataset and 0.94 in the external other-vendor test-dataset, although the models with the higher sensitivity also had higher false positive rates (see Table 4). Between the different pre-processing pipelines, there was a statistically significant difference between the false positive rate for the different voxel sizes, with a voxel size of 0.5 × 0.5 × 0.5 mm yielding up to seven times more FPs/case on the external same-vendor test-dataset (e.g. using the pre-processing pipeline with the highest DSC: 0.87 ± 1.35 vs. 6.56 ± 5.43 FPs/case, p < 0.001).

**Table 4:** Results of Dice score coefficient (*DSC*), false positives per case (*FPs/case*), and aneurysm-level sensitivity without any pre-processing and for the three pre-processing methods.

Pre-processing approach	External same-vendor test dataset				External other-vendor test dataset			
	DSC	FPs/case	Sensitivity	Specificity	DSC	FPs/case	Sensitivity	Specificity
No preprocessing	0.31 ± 0.25	47.6 ± 82.75	0.94	0.04	0.06 ± 0.17	3221 ± 6081	0.89	0.04
N4 bias field correction + resampling (1 × 1x1 mm) + intensity normalization	0.56 ± 0.24	1.96 ± 1.78	0.97	0.08	0.62 ± 0.27	2.46 ± 3.23	0.86	0.18
N4 bias field correction + resampling (0.5 × 0.5x0.5 mm) + intensity normalization	0.57 ± 0.23	4.51 ± 3.60	0.98	0.05	0.58 ± 0.28	6.03 ± 10.21	0.89	0.06
N4 bias field correction + resampling (1 × 1x1 mm) + cropping + intensity normalization	0.60 ± 0.25	0.87 ± 1.35	0.97	0.38	0.65 ± 0.26	0.96 ± 2.38	0.92	0.53
N4 bias field correction + resampling (0.5 × 0.5x0.5 mm) + cropping + intensity normalization	0.51 ± 0.25	6.56 ± 5.43	0.97	0.01	0.57 ± 0.23	5.90 ± 7.94	0.94	0.08
“Sichtermann B + N4: 1 mm” Intensity normalization + resampling (1 × 1x1 mm) + BET2 brain extraction + N4 Bias field correction	0.57 ± 0.25	0.99 ± 1.31	0.94	0.27	0.52 ± 0.32	1.12 ± 2.04	0.78	0.33
“Sichtermann B + N4: 0.5 mm” Intensity normalization + resampling (0.5 × 0.5x0.5 mm) + BET2 brain extraction + N4 Bias field correction	0.50 ± 0.28	4.73 ± 5.10	0.89	0.02	0.54 ± 0.27	2.71 ± 3.06	0.86	0.10

### Influence of scanner and aneurysm features on aneurysm segmentation

**Figure 6:**
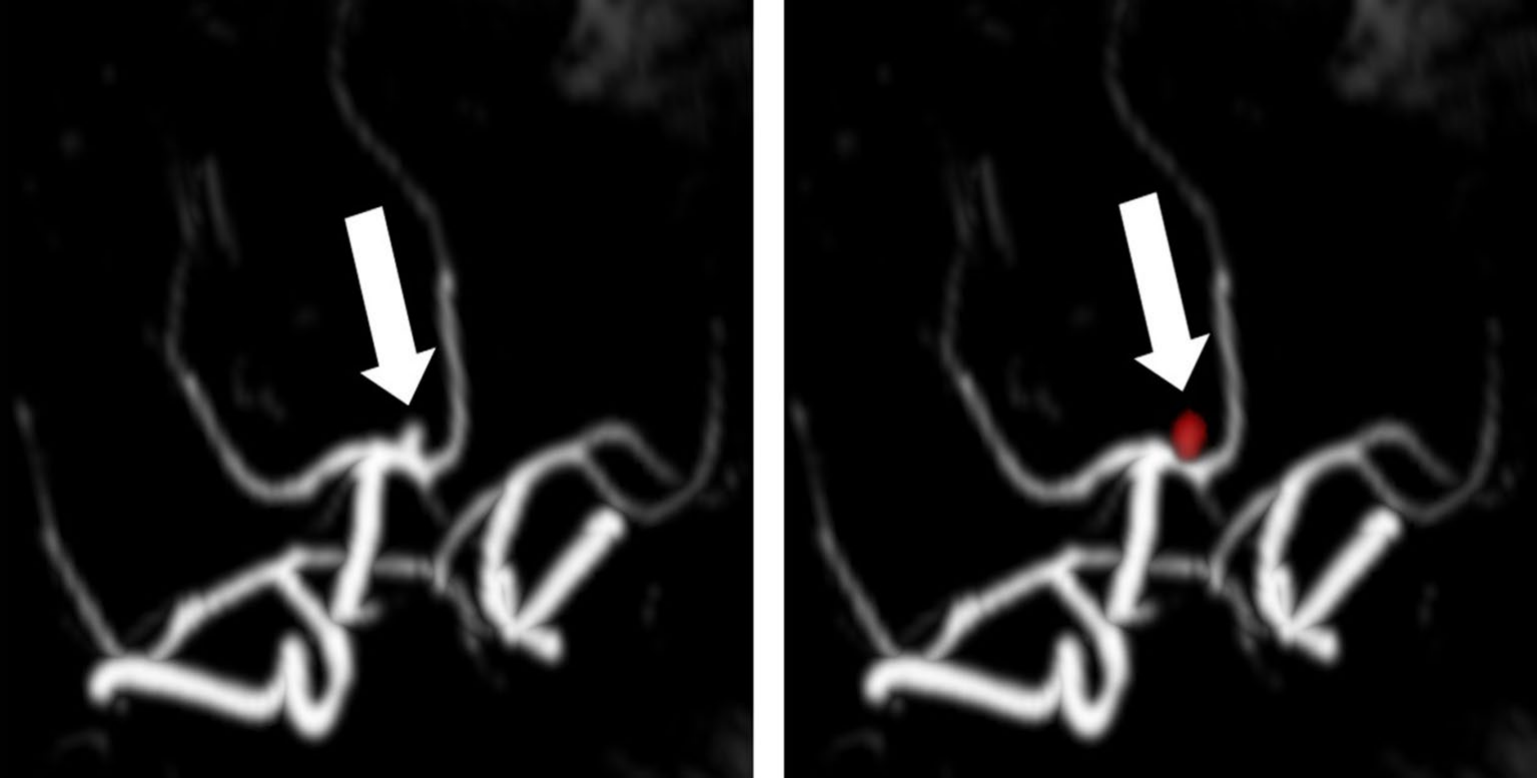
Example of a P1-aneurysm in a TOF-MRA (arrow) with the segmentation of our algorithm overlayed in the right image from the model yielding the highest dice similarity coefficient (bias field corrected, 1 × 1x1 mm voxel size, off-center cropped; TOF-MRA from the external same-vendor test-dataset acquired on a Siemens Avanto 1.5T MRI system).

**Figure 7:**
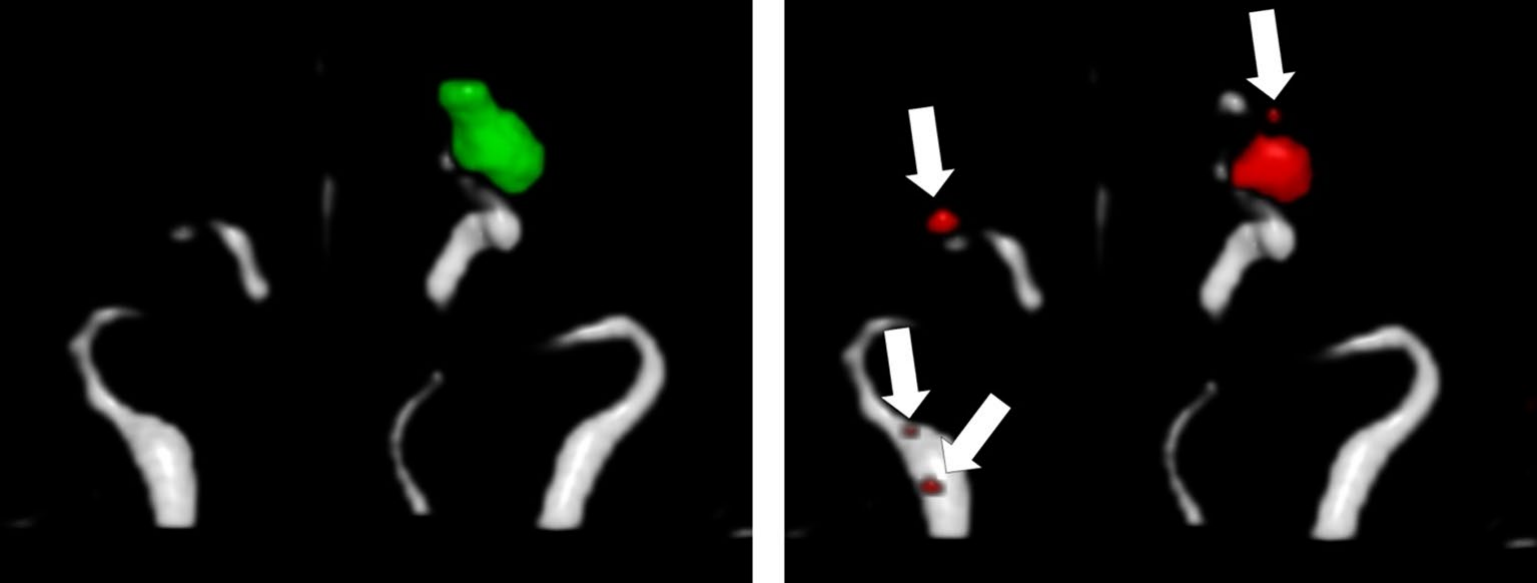
Example of an aneurysm segmentation with a lot of false positives from the model yielding the highest dice similarity coefficient (bias field corrected, 1 × 1x1 mm voxel size, off-center cropped). The contrast in the TOF-MRA is insufficient, which likely led to the poor segmentation (TOF-MRA from the external same-vendor test-dataset acquired on a Siemens Symphony 1.5T MRI system).

For the model with the highest DSC on the external datasets, there was no statistically significant difference in the DSC between examinations acquired on 1.5-Tesla and 3-Tesla magnets on the same-vendor test-dataset (0.66 ± 0.24 vs. 0.55 ± 0.33, p = 0.46). The location of the aneurysms showed no significant influence on the accuracy of the segmentation in the external same-vendor test-dataset (p = 0.52, see Table 3). A weak, but statistically significant correlation between the total aneurysm volume and the DSC (r = 0.34, p = 0.04) was found in the sense that the model achieved higher DSCs on larger aneurysms. There was a strong correlation between the total aneurysm volume predicted by our model and the manually segmented ground-truth volume (r = 0.89, p < 0.001 for the same-vendor test data and r = 0.91, p < 0.001 for the other-vendor test data). The mean absolute error in the same-vendor test dataset was 32 ± 60 mm^3^ (IQR 5–25 mm^3^, 120 ± 149 mm^3^ ground truth aneurysm volume) and in the other-vendor test dataset 61 ± 101 mm^3^ (IQR 11–63 mm^3^, 214 ± 269 mm^3^ ground truth aneurysm volume). Figures 6, 7 show examples of an excellent segmentation result and the case with the largest number of false positives after pre-processing.

### Comparison with the previous publication

The experiment closely replicating the previous pre-processing pipeline at a voxel size of 0.5 × 0.5 × 0.5 mm was also tested on the external test-datasets, a key aspect not explored in the previous publication. It achieved a sensitivity of 0.89 and 0.86 in the external same-vendor and external other-vendor test-dataset, respectively, a DSC of 0.50 ± 0.28 and 0.54 ± 0.27, and a false positive rate of 4.73 ± 5.10 and 2.71 ± 3.06, which is comparable to previously published results using the full proposed pre-processing without post-processing (pipeline “D0”, using an internal test-dataset): A sensitivity of ~ 0.86, a DSC of 0.53 ± 0.31, and a false positive rate of ~ 2.7 FPs/case^8^.

Compared to the best results of the previous publication across the different proposed pre- and post-processing pipelines (sensitivity of up to 0.9 (pipeline A0), DSC of up to 0.53 ± 0.29 (pipeline B0) and a false-positive rate of 0.8 FPs/case (pipeline D7)), our best performing approach achieved an improved sensitivity (0.97 and 0.94 in the external same-vendor and other vendor test-datasets, respectively), a higher DSC (0.60 ± 0.25 and 0.65 ± 0.26), and a comparable false-positive rate (0.87 ± 1.35 and 0.96 ± 2.38 FPs/case) using a single pipeline without any post-processing. Specificity could not be compared, since the previous publication only included patients with aneurysms.

### Transferability between MRI vendors

To test the transferability of the segmentation model trained and tested on images of a single MRI vendor to other vendors, we compared the segmentation results of our best performing model (1 × 1 × 1 mm, cropped data) in the external same-vendor test-dataset to the performance in the external other-vendor test-dataset. There was no statistically significant difference between the DSC of both external test-datasets (0.60 ± 0.25 vs. 0.65 ± 0.26; p = 0.49). The sensitivity was also comparable (0.97 vs. 0.92, p = 1). The number of false positives per patient also showed no statistically significant difference (0.87 ± 1.35 FP/case vs. 0.96 ± 2.38 FP/case, p = 0.80). The specificity was better in the external other-vendor test-dataset (0.53) than in the external same-vendor and test-dataset (0.38).

## Discussion

In the current study, we successfully replicated the core principles of a deep learning approach for aneurysm segmentation in TOF-MRAs^8^, while at the same time proposing a novel, more robust, and fully automatable pre-processing. The best-performing pipeline and model demonstrated excellent generalizability and transferability in two independent external test-datasets, consisting of TOF-MRAs with and without aneurysms, without any need for post-processing. Pre-processing was found to be indespensable to lower the false-positive rate.

Subarachnoid hemorrhage due to aneurysm rupture is often life-altering and potentially fatal. Reliable diagnosis of unruptured aneurysms is beneficial and enables optimal management and treatment^18^. However, non-specialists, and to a lesser degree even experienced neuroradiologists, often have limited sensitivity in detecting incidental aneurysms, particularly small ones^4^. Automatic detection has been shown to significantly improve aneurysm detection^10^. We decided to replicate and improve on the core principles of a previous study using the deep learning framework “DeepMedic”, since the study achieved very promising results without relying on complex pre-processing, such as vessel segmentation^9,19^, and employed an established and well-tested, openly available framework.

Compared to the previous publication, we improved segmentation accuracy and sensitivity, which is attributable to the larger training cohort (n = 235 vs. n = 58) and different approaches to pre-processing. In the previous publication, all TOF-MRA scans were resampled to a voxel size of 0.5 × 0.5 × 0.5 mm. However, our study indicates that the false positive rate of a model trained using a voxel size of 1 × 1 × 1 mm is significantly decreased, likely due to a higher signal-to-noise ratio. The false-positive rate in the external same-vendor test-dataset is comparable to the best results of the previous publication, and slightly worse in the external other-vendor test-dataset. This is very likely attributable to the nature of the experiment, since the other-vendor test-dataset only included images from vendors other than the training and external same-vendor test-dataset. The previous publication employed post-processing to lower the false positive rate (from over 6 FPs/case to 0.8 FPs/case). This, however, was followed by a decrease in sensitivity (from 0.9 to 0.79). Our approach achieved a high sensitivity (0.97 and 0.95 on the test-datasets) and a low false positive rate without any post-processing.

In general, our findings indicated lower specificity compared to other research available in the literature^20^. However, it’s important to highlight that reliable specificity metrics are lacking in the literature, frequently leaving it unclear whether studies are reporting specificity at the patient level or based on smaller segments (“patches”) of the complete images. Joo et al. reported a patient-wise specificity of 94%^20^. However, it is worth noting that the training and test-dataset were acquired at the same institution, whereas we rigorously tested on external data only to assess real-world performance. Still, we intend to conduct further studies to enhance the specificity.

In the context of AI-assisted aneurysm detection, a high sensitivity, especially for small, easy to miss aneurysms, is arguably more crucial than a low false positive rate, although a sensible balance has to be achieved in a routine clinical setting to not impede the effectiveness of a tool due to the manual verification of too many falsely detected aneurysms.

We also deviated from the previous study by proposing different pre-processing methods. We changed the order of the pre-processing to start with the bias field correction using the whole image volume, since the bias field applies to the whole image, and concluded each pre-processing with the intensity normalization. We swapped skull stripping using FSL’s BET2 for a simple off-center cropping approach. BET2 is prone to remove parts of interest from the image volume, particularly at the skull base, where many aneurysms are located. This may explain why Sichtermann et al. needed to manually adjust the fractional intensity threshold for each image volume^8^, which is impractical for fully automated aneurysm tools and led us to propose a simple, robust, automatable, and effective off-center cropping of the TOF-MRAs.

While our best performing model has yielded a reasonably high DSC and a strong correlation between total predicted and ground-truth aneurysm volume, an exact aneurysm segmentation may not be essential in a clinical setting. A coarse segmentation could be sufficient for detection and verification of an aneurysm location. When segmentation is merely used to obtain the location of the aneurysm, i.e. the task at hand is aneurysm detection, a well-balanced sensitivity and false positive rate are much more essential.

We expected that larger aneurysms are more easily detectable and accurately segmented. However, there was only a weak correlation between the aneurysm volume of the patient and the accuracy of segmentation. While Sichtermann et al. also found a difference in the DSC based on aneurysm size, Claux et al., who used a two-stage U-Net Deep Learning network, did not find a significant correlation between aneurysm size and segmentation accuracy^9^.

Studies investigating the robustness and replicability of machine learning experiments in radiology are rare and mostly focused on radiomics^21,22^. Few studies have examined the replicability or transferability of deep learning models across different image acquisition environments^23^. Our study indicates that a neural network trained on MRI images from one vendor at one institution can generalize to images from other institutions and scanners with different sequences, parameters and contrast. This is an encouraging finding for deploying deep learning models in the real world.

### Limitations

Our work has some limitations. While the previous publication extensively studied different pre- and post-processing approaches, we adopted a more “minimal” and automatable approach to pre-processing and forwent post-processing, which we believe led to our robust results across the external test-datasets. Extending the previous publication’s experiments, we also resampled the images to a voxel size of 1 × 1 × 1 mm, instead of only 0.5 × 0.5 × 0.5 mm, which was primarily driven by 62% of our raw real-world training data featuring a voxel size larger than 0.5 × 0.5 × 0.5 mm in at least one dimension. Therefore, it might be possible that our best-performing approach could miss very small aneurysms, which should be further evaluated on a larger sample of such very small aneurysms. Furthermore, our replication experiment did not fully replicate the previous publication’s pre-processing pipeline, since we chose not to manually adjust each brain extraction. Nevertheless, we combined all proposed pre-processing steps, with automated brain extraction, and achieved very similar results to the previous publication. Finally, the other-vendor test dataset is heterogeneous, comprising various MRI scanner vendors and scanner models, which complicates the comparability of the results. Nonetheless, our results demonstrate that our approach is effective even in this setting and despite of the model being trained only on images from a single MRI vendor.

## Conclusion

The current study successfully replicated the core principle of a previous study to detect and segment unruptured cerebral aneurysm in TOF-MRAs. By introducing a novel, more robust and automatable preprocessing pipeline combined with a larger training dataset, we were able to improve both detection and segmentation of cerebral aneurysms. We stringently tested the model on an external same-vendor test-dataset, using data from other hospitals acquired on MRI scanners from the same vendor as used in the training dataset, and successfully demonstrated robust transferability in an external other-vendor dataset with equally good results both in patients with and without aneurysms, which is an encouraging finding for the real-world deployment of such models.

## Data Availability

The datasets generated during and/or analyzed during the current study are available from the corresponding author upon reasonable request.

## Abbreviations

TOF-MRA: Time of flight magnetic resonance angiogram
PACS: Picture achieving and communication system
DICOM: Digital imaging and communications in medicine
NifTI: Neurimaging informatics technology initiative
FSL: FMRIB software library
BET2: Brain extraction tool
CNN: Convolutional neural network
DSC: Dice score coefficient
FP: False positive
CC: Connected component
